# Evaluating the hepatoprotective, ameliorative and antioxidant potentials of the crude aqueous leafy extracts of *Mangifera indica* plant against acute paracetamol-induced hepatotoxicity in a mouse model

**DOI:** 10.2144/fsoa-2021-0119

**Published:** 2022-06-17

**Authors:** Fidelis Azagbor Ilukho, Olumuyiwa John Fasipe, Flora Ruth Aigbe

**Affiliations:** 1 Department of Pharmacology & Therapeutics, Faculty of Basic Clinical Sciences, College of Medicine, Edo University, Iyamho, Edo state, Nigeria; 2 Department of Pharmacology & Therapeutics, Faculty of Basic Clinical Sciences, College of Medicine, University of Medical Sciences, Ondo City, Ondo State, Nigeria; 3 Department of Pharmacology, Therapeutics & Toxicology, Faculty of Basic Medical Sciences, College of Medicine, University of Lagos, Idi-Araba, Mushin, Lagos State, Nigeria

**Keywords:** acute acetaminophen/paracetamol-induced hepatotoxicity, ameliorative, antioxidant, crude aqueous leafy extract, effects, hepatoprotective, *Mangifera indica* plant

## Abstract

**Background:**

Drug-induced hepatotoxicity is a major public health issue of concern. It significantly affects the development of new pharmaceutical drugs and has led to the withdrawal of many promising pharmaceutical drugs from the pharmaceutical market.

**Aim:**

The aim of this study was to evaluate the hepatoprotective, ameliorative and antioxidant effects of the crude aqueous leafy extract of *Mangifera indica* plant and its different separating medium fractions against acute acetaminophen (paracetamol)-induced hepatotoxicity in a mouse model.

**Methods & materials:**

Twelve different groups of six mice (three males and three females) were used for this study. Acetaminophen at a single lethal hepatotoxic dose of 3 g/kg was orally administered on the seventh day to the mice in groups 2 to 12 after their 6-day pretreatment duration for the induction of hepatotoxicity; and were then left for 24 hours before the collection of specimen samples were completed, while group 1 served as control.

**Results:**

The crude aqueous leafy extract of *M. indica* (125-250 mg/kg) produced a dose-dependent reversal of the lethal hepatotoxic effect of oral 3 g/kg dose of paracetamol. At the dose of 250 mg/kg, it significantly (p < 0.0001) reduced the levels of hepatic enzymes markers (alanine transaminase [ALT], aspartate transaminase [AST] and alkaline phosphatase [ALP]) in the serum of treated animals. Also, the effects of the crude aqueous leafy extract were found to be statistically significant (p < 0.0001) more than that of its different separating medium fractional components.

**Conclusion:**

The findings from this study demonstrated that the crude aqueous leafy extract of *M. indica* possesses hepatoprotective effect, possibly mediated through the induction of antioxidant enzymes to prevent the occurrence of oxidative stress damage or most likely through the inhibition of pro-inflammatory mediators which are being induced by the lethal hepatotoxic dose of paracetamol.

## Plain language summary

The findings from this study demonstrated that the crude aqueous leafy extract of *Mangifera indica* possesses a hepatoprotective effect, possibly mediated through the induction of antioxidant enzymes to prevent the occurrence of oxidative stress damage or most likely through the inhibition of proinflammatory mediators which are being induced by the lethal hepatotoxic dose of paracetamol.

In spite of the fact that so much is known about drug-induced hepatotoxicity, it is still a major public health issue of major concern. The challenges posed by this pathological state affect mainly the development of new pharmaceutical drugs and the withdrawal of promising pharmaceutical drugs from the pharmaceutical market. According to the report by Niknahad *et al.*, troglitazone was introduced as a promising antidiabetic drug but had to be withdrawn from market within a few years because of serious hepatic (liver) injury accompanied with its administration [1,2]. It appears that its sulfate conjugate inhibits bile salt transport from hepatocytes, leading to severe idiosyncratic hepatotoxicity [1–3].

Hepatotoxicants can be divided into two groups; toxicants that directly affect the liver (intrinsic hepatotoxicity) and toxicants that mediate an immune response (hypersensitivity) or idiosyncratic hepatotoxicity. Intrinsic or direct hepatotoxicity is usually dose-dependent, reproducible in different animal models and has a consistent period of onset. For example the acetyl-para-aminophenol (APAP)-induced hepatotoxicity is a dose-dependent hepatotoxicity [4]. Idiosyncratic drug-induced hepatotoxicity comprises of several factors including, but not limited to, lack of reproducible animal models, dose-independent and host-dependent, immune system or genetic variability [5,6]. Examples of idiosyncratic hepatotoxins are troglitazone and chlorpromazine [7,8]. The mechanisms of action of the hepatotoxicant agents are known but are not totally defined. Some of the mechanisms of action of hepatotoxicants include; directly action on the critical cellular system like plasma membrane, mitochondria, endoplasmic reticulum, nucleus and lysosomes, thus disrupting their activity. Various hepatotoxicants bind to mitochondrial membranes and enzymes, disrupting energy metabolism and cellular respiration [9]. Many hepatotoxicants act as direct inhibitors and uncouplers of mitochondrial electron transport chain [10–17]. Other popular mechanisms of action include lipid peroxidation and redox recycling and disruption of calcium homeostasis [17].

All hepatotoxins produce a wide variety of clinical and histopathological features of hepatic injury. Liver injury can be diagnosed by the concentration levels of certain biochemical markers such as alanine aminotransferase (ALT), aspartate aminotransferase (AST), alkaline phosphatase (ALP), gamma glutamyl transferase and bilirubin levels [12,16,18–20]. Elevations in serum enzyme levels are taken as the relevant indicators of hepatic toxicity whereas increases in both total and conjugated bilirubin levels are measures of overall liver function [12,16,18–20]. An elevation in transaminase levels in conjunction with a rise in bilirubin level to more than double its normal upper limit is considered as an ominous marker for hepatotoxicity [18].

Hepatotoxicity can be characterized into two main groups, each with a different mechanism of injury: hepatocellular and cholestatic [19]. Hepatocellular or cytolytic injury involves predominantly initial serum aminotransferase level elevations, usually preceding increases in total bilirubin levels and modest increases in ALP levels while cholestatic injury is characterized by predominantly initial ALP level elevations that precede or are relatively more prominent than increases in the levels of serum aminotransferases. Generally; mixed type of injuries, involving both hepatocellular and cholestatic mechanisms, occurs [20].

The ratio value of ALT to ALP plays an important role in deciding the type of liver damage by hepatotoxins. The ratio is greater than or equal to five (≥5) during predominant hepatocellular damage while the ratio is less than or equal to two (≤2) during predominant cholestatic liver damage. During mixed type of liver damage, the ratio ranges between two and five. ALT, AST and in combination with total bilirubin are primarily recommended for the assessment of hepatocellular injury in rodents and non rodents in nonclinical studies. ALT is considered a more specific and sensitive indicator of hepatocellular injury than AST; because, for AST level to be elevated, there must be mitochondrial membrane damage which is located intracellular to the cell/plasma membrane. But in contrast, ALT is exclusively a cytoplasmic enzyme which can easily leak-out into the systemic circulation following damage to the hepatocytes’ cell/plasma membranes [20,21].

Herbal remedies have shown effective recourse to the tolls of hepatotoxic agents. Out of the many herbs and herbal drugs which are reported to have hepatoprotective effect [22–26], only four plants have been elucidated scientifically following internationally accepted standard protocols to develop evidence based alternative herbal hepatoprotective drugs [25,26]. Silymarin, a flavonolignan from *Silybum marianum* (milk thistle) is an effective herbal hepatoprotective agent which prevents damage to the liver by antioxidative, antilipid peroxidative, anti-inflammatory, membrane stabilizing, immunomodulatory and liver regenerating mechanism [22–25,27]. The medical therapeutic recommended dose of oral Silymarin is usually 600 mg/day which is being administered 200 mg three-times daily per os. The 200 mg milk thistle plant extract usually contains 140 mg of silymarin (that is equivalent to 70% silymarin content); therefore, this study will use a silymarin oral daily dose of 10 mg/kg for the pre-treatment of some mice in a particular group [22–25,27]. *Glycyrrhiza glabra*, *Picrorhiza Kurroa* and *Phyllanthus amarus* have also been proved scientifically to possess hepatoprotective effect [25,26].

The search for newer efficacious hepatotoprotective agents is still on; which is the basis for which we intend to evaluate the hepatotoprotective effect of the crude aqueous leafy extract of *Mangifera indica* (commonly known as mango plant) in a mouse model using internationally accepted standard scientific protocol. Furthermore, the aim and objective of this study is to investigate the hepatoprotective, ameliorative and antioxidant effects of the crude aqueous leafy extract of *M. indica* plant and its different separating medium fractions against acute acetaminophen (paracetamol)-induced hepatotoxicity in a mouse model.

## Materials & methods

### Plant collection & identification

The fresh *M. indica* leaves were collected from a residential area by the researchers. This fresh leafy specimen of *M. indica* was identified by an experienced botanist (OO Oyebanji) at the Department of Botany, University of Lagos (Akoka-Yaba, Nigeria). The assigned identification number was LUH 7273. The leafy specimen was deposited in the herbarium of the University of Lagos.

### Extract preparation

The authenticated leafy specimen was clean and shade-dried for more than 2 weeks before being ground into powder. The powdery leaf was weighed and approximately (300 g) of the powdered leaf was soaked in 2000 ml of distilled water for 24 h with occasional shaking and stirring inside a 5 l beaker. The extracts were separated from the residues by filtering through several layers of muslin cloth for coarse filtration. The residues were extracted with different solvents namely the fractionated extract, ethyl acetate extract and petroleum ether-chloroform extract. These filtered extracts were concentrated and solvents were evaporated under reduced pressure at 40°C, using a rotary evaporator (EYELA, CA-1111, Rikakikai Company Ltd, Tokyo, Japan). The dried concentrated extracts were weighed to calculate the percentage yield and stored in a refrigerator at minus -8°C, until needed for analyses [26,28–32].

### Laboratory animals

The apparently healthy mice of both sexes (weight between 20 and 25 g) used for this study were obtained from the Animal Laboratory Centre (ALC [College of Medicine University of Lagos (CMUL), Lagos, Nigeria]). The mice were kept in a well-ventilated environment, housed in standard cages and fed with standard rodent feed and had free access to water *ad libitum*. The mice model was acclimatized for 14 days before commencing the experiment.

### Acute toxicity profile

The acute toxicity profile for the crude aqueous leafy extract of *M. indica* was determined by using the guideline methods originally described by the Organization for Economic Co-operation and Development (OECD) in the year 2000 in Paris, France for acute oral toxicity testing. The mice within the five different experimental groups used for acute oral toxicity testing were fasted for 12 h before receiving administration of the crude aqueous leafy extract of *M. indica* plant up to the dose of 5000 mg/kg orally, while the mice within the control group were fasted for 12 h before receiving administration of 10 ml/kg of distilled water (placebo) orally. The crude aqueous leafy extract was administered to the mice within the five different experimental groups (with each group comprising of five mice per group) at the oral dose of 5 mg/kg (for each of the mice within the first group), 50 mg/kg (for each of the mice within the second group), 300 mg/kg (for each of the mice within the third group), 2000 mg/kg (for each of the mice within the fourth group) and 5000 mg/kg (for each of the mice within the fifth group), respectively to determine the acute median lethal dose (LD_50_) of the plant extract among 50% of the mice through the oral route, but there was not any observed mortality up to the dose of 5000 mg/kg orally. All the mice were closely observed for toxic symptoms during the first 2 h and then for 24 h post administration.

The crude aqueous leafy extract of *M. indica* was observed to have a median effective dose (ED_50_) of 250 mg/kg, which was the minimum effective dose that produced an observed significant hepatoprotective, ameliorative and antioxidant effects in at least 50% of the mice used for the acute acetaminophen (paracetamol)-induced hepatotoxicity testing.

The therapeutic index [TI] value for the crude aqueous leafy extract of *M. indica* was determined using the formulae below [26–29,33]:

$$\text{Therapeutic Index}[\text{TI}]=\frac{\text{Median Lethal Dose }[{\text{LD}}_{50}]}{\text{Median Effective Dose }[{\text{ED}}_{50}]}$$

### Grouping of experimental animals

Twelve different groups of six mice each (three males and three females) were used for this study. The grouping follows this arrangement:

- Group 1 (control) was administered oral 10 ml/kg of normal saline for 6-day pretreatment duration.
- Group 2 (acetaminophen-treated group) was administered oral 10 ml/kg of normal saline for 6-day pretreatment duration before the induction of hepatotoxicity with a single oral paracetamol dose of 3 g/kg on the 7th day.
- Group 3 was administered oral 10 ml/kg of olive oil for 6-day pretreatment duration before the induction of hepatotoxicity with a single oral paracetamol dose of 3 g/kg on the 7th day.
- Group 4 was administered oral 10 mg/kg of silymarin for 6-day pretreatment duration before the induction of hepatotoxicity with a single oral paracetamol dose of 3 g/kg on the 7th day.
- Group 5 was administered oral 125 mg/kg of crude aqueous leafy extract of *M. indica* for 6-day pretreatment duration before the induction of hepatotoxicity with a single oral paracetamol dose of 3 g/kg on the 7th day.
- Group 6 was administered oral 250 mg/kg of crude aqueous leafy extract of *M. indica* for 6-day pretreatment duration before the induction of hepatotoxicity with a single oral paracetamol dose of 3 g/kg on the 7th day.
- Group 7 was administered oral 125 mg/kg of fractionated aqueous leafy extract of *M. indica* for 6-day pretreatment duration before the induction of hepatotoxicity with a single oral paracetamol dose of 3 g/kg on the 7th day.
- Group 8 was administered oral 250 mg/kg of fractionated aqueous leafy extract of *M. indica* for 6-day pretreatment duration before the induction of hepatotoxicity with a single oral paracetamol dose of 3 g/kg on the 7th day.
- Group 9 was administered oral 125 mg/kg of ethyl acetate fraction of the leafy extract of *M. indica* for 6-day pretreatment duration before the induction of hepatotoxicity with a single oral paracetamol dose of 3 g/kg on the 7th day.
- Group 10 was administered oral 250 mg/kg of ethyl acetate fraction of the leafy extract of *M. indica* for 6-day pretreatment duration before the induction of hepatotoxicity with a single oral paracetamol dose of 3 g/kg on the 7th day.
- Group 11 was administered oral 125 mg/kg of petroleum ether-chloroform fraction of the leafy extract of *M. indica* for 6-day pretreatment duration before the induction of hepatotoxicity with a single oral paracetamol dose of 3 g/kg on the 7th day.
- Group 12 was administered oral 250 mg/kg of petroleum ether-chloroform fraction of the leafy extract of *M. indica* for 6-day pretreatment duration before the induction of hepatotoxicity with a single oral paracetamol dose of 3 g/kg on the 7th day.

### Hepatotoxicity induction

After the 6-day pretreatment duration; on the 7th day, a single oral 3 g/kg lethal hepatotoxic dose of paracetamol was administered to all the animals in the groups 2–12 for the induction of hepatotoxicity; and were then left for 24 h before the collection of specimen samples were done.

### Collection of specimen samples

Blood samples were collected using cardiac puncture. The animals were anesthetized with chloroform so that they can be opened up using dissecting set, in order to collect blood samples from the beating hearts of the animals into the plain sample bottle for blood chemistry. The collected blood samples were centrifuged at 3000 rpm for 15 min in order to separate the serum from the blood cells. The supernatants now known as the sera were poured into another plain sample bottle, and then frozen for liver function test and lipid profile in the laboratory. The liver organ specimens of the animals from each of the twelve groups were collected using universal sample bottles, and then being preserved inside iced phosphate buffer solution for *in vivo* antioxidant assay.

### Phytochemical screening analysis

Qualitative phytochemical screening analysis of the crude aqueous leafy extract of *M. indica* was carried out using internationally accepted standard procedures and tests previously mentioned by Sofowora (1993), to reveal the presence of chemical constituents such as alkaloids, flavonoids, tannins, terpenoids, saponins, anthraquinones, reducing sugars, cardiac glycosides, steroids, phenols and phlobatanin among others [21,34]. Quantitative estimation of the total amount of each detectable phytochemical constituents of the crude aqueous leafy extract was also evaluated [21,34].

#### Qualitative phytochemical screening analyses methods

These qualitative phytochemical screening analyses methods employed during this study are described below in (1) to (7) [21,34]:

**(1) Test for alkaloid**

5 ml sample of the extract was dissolved in 3ml acidified ethanol and warmed slightly afterwards was filtered. Few drops of Mayer’s reagent and 1ml of Dragendroff’s reagent were added to 1ml of the filtrate and turbidity was observed [21,34].

**(2) Test for tannins**

Few drops of 0.1% ferric chloride was added to the extract solutions and observed for brownish-green or blue-black coloration, which signified the presence of tannins [21,34].

**(3) Test for flavonoids**

Three methods were used to determine the presence of flavonoids in the plant sample;

(a) 5 ml of dilute ammonia solution was added to 5 ml of the extract solutions followed by the addition of concentrated H2SO4. A yellow coloration observed in each extract indicated the presence of flavonoids. The yellow coloration disappeared on standing [21,34].

(b) Few drops of 1% aluminium solution were added to a portion each filtrate. A yellow coloration was observed indicating the presence of flavonoids [21,34].

(c) A portion of the powered plant sample was in each case heated with 10ml of ethyl acetate over a stream bath for 3min. the mixture was filtered and 4ml of the filtrate was shaken with 1ml of dilute ammonia solution. A yellow coloration was observed indicating a positive test for flavonoids [21,34].

**(4) Test for cardiac glycosides (Keller–Killiani test)**

5 ml of plant sample was treated with 2 ml of glacial acetic acid containing one drop of ferric chloride solution. This was underplayed with 1ml of concentrated sulphuric acid. A brown ring of the interface indicates a deoxysugar characteristic of cardenolides. A violet ring may appear below the brown ring, while in the acid layer, a greenish ring may form just gradually throughout the thin layer [21,34].

**(5) Test for steroids**

2 ml of acetic anhydride was added to 0.5 g ethanolic extract of each sample with 2 ml H2S04. The color changed from violet to blue or green in some samples indicating the presence of steroids. Test for terpenoids (Salkowski test) 5ml of each extract was mixed in 2 ml of chloroform, and concentrated H2S04 (3 ml) was carefully added to form a layer. A reddish brown coloration of the interface was formed to show positive results for the presence terpenoids [21,34].

**(6) Test for saponin**

The powered sample of 2 g was boiled in 20 ml of distilled water bath and filtered. 10 ml of the filtrate was mixed with 5 ml of distilled water and shaken vigorously for a stable persistent froth. The frothing was mixed with 3 drops of olive oil and shaken vigorously, then observed for the formation of emulsion [21,34].

**(7) Test for phlobatanin**

Deposition of red precipitate when an aqueous extract of each plant sample was boiled with 1% aqueous hydrochloric acid was taken as evidence for the presence of phlobatanins [21,34].

#### Quantitative Determination of Phytochemical Constituents

The various methods and techniques used for the quantitative determination of phytochemical constituents of the crude aqueous leafy extract of *M. indica* plant are described below in (a) to (e) [21,34]:

**(a) Determination of total phenols by spectrophotometric method**

The fat free sample was boiled with 50 ml of ether for the extraction of the phenolic component for 15 min. 5 ml of the extract was pipetted into a 50 ml flask, then 10 ml of distilled water was added. 2 ml of ammonium hydroxide solution and 5 ml of concentrated amylalcohol were also added. The samples were made up to mark and left to react for 30 min for colour development. This was measured at 505 nm wavelength [21,34].

**(b) Alkaloid determination using Harborne method**

Of the sample, 5 g was weighed into a 250 ml beaker and 200 ml of 10% acetic acid in ethanol was added and covered and allowed to stand for 4 h. This was filtered and the extract was concentrated on a water bath to one-quarter of the original volume. Concentrated ammonium hydroxide was added dropwise to the extract until the precipitation was complete. The whole solution was allowed to settle and the precipitated was collected and washed with dilute ammonium hydroxide and then filtered. The residue is the alkaloid, which was dried and weighed [21,34].

**(c) Tannin determination by Van-Burden and Robinson method**

Of the sample, 500 mg was weighed into a 50 ml plastic bottle. 50 ml of distilled water was added and shaken for 1 h in a mechanical shaker. This was filtered into a 50 ml volumetric flask and made up to the mark. Then 5 ml of the filtered was pipetted out into a test tube and mixed with 2 ml of 0.1 M FeCl3 in 0.I N HCl and 0.008 M potassium ferrocyanide. The absorbance was measured at 120 nm within 10 min [21,34].

**(d) Saponin determination**

The method used was that of Obadoni and Ochuko. The samples were ground and 20 g of each were put into a conical flask and 100 cm^3^ of 20% aqueous ethanol were added. The samples were heated over a hot water bath for 4 h with continuous stirring at about 55°C. The mixture was filtered and the residue re-extracted with another 200 ml 20% ethanol. The combined extracts were reduced to 40 ml over water bath at about 90°C. The concentrate was transferred into a 250 ml separator funnel and 20 ml of diethyl ether was added and shaken vigorously. The aqueous layer was recovered while the ether layer was discarded. The purification process was repeated. 60 ml of n-butanol was added. The combined n-butanol extracts were washed twice with 10 ml of 5% aqueous sodium chloride. The remaining solution was heated in a water bath. After evaporation, the samples were dried in the oven to a constant weight; the saponin content was calculated as percentage [21,34].

**(e) Flavonoid determination by Bohm and Kocipai-Abyazan method**

Of the plant sample, 10 g was extracted repeatedly with 100 ml of 80% aqueous methanol at room temperature. The whole solution was filtered through whatman filter paper no. 42 (125 mm). The filtrate was later transferred into a crucible and evaporated into dryness over a water bath and weighed to a constant weight [21,34].

### Total antioxidant capacity determination

The total antioxidant capacity of the extract was determined using the method of Prieto *et al.*. A sample of the extract (0.3 ml) was mixed with 3 ml of antioxidant reagent solution (0.6 m sulphuric acid, 28 mm sodium phosphate and 4 mm ammonium molybdate). The tubes were capped and incubated in a boiling water bath at 95°C for 90 min. After the samples had cooled down to room temperature, the absorbance of the aqueous solution of each was measured at 695 nm. The total antioxidant capacity was expressed as equivalent to ascorbic acid [21,34].

### Lipid peroxidation assay

Lipid peroxidation was induced by ferrous (Fe^2+^) coupled-ascorbate system in liver homogenate and estimated as thiobarbituric acid reacting substances by the method of Buege and Aust. Freshly excised rat liver was sliced and processed to obtain 10% homogenate in cold 150 mM KCl-Tris-HCl buffer. The reaction mixture contained liver homogenate, Tris-HCl buffer (20 mM pH 7.0), FeCl_2_ (2 mM), ascorbic acid (10 mM) and 0.5 ml plant extract (25–100 μg/ml) in a final volume of 1 ml. The reaction mixture was incubated at 37°C for 1 h. Lipid peroxidation was measured as malondialdehyde (MDA) equivalent using trichloroacetic acid (TCA), thiobarbituric acid (TBA) and HCl (TBA-TCA reagent: 0.375% w/v TBA; 15% w/v TCA and 0.25 n HCl). The incubated reaction mixture was mixed with 2 ml of TBA-TCA reagent and heated in a boiling water bath for 15 min. After cooling, the flocculent precipitate was removed by centrifugation at 10,000 × g for 5 min. Finally, MDA concentration in the supernatant fraction was determined spectrophotometrically at 535 nm. The concentrations of extract that would cause 50% inhibition of the production of thiobarbituric acid reactive substances (IC_50_ values) were calculated. Ascorbic acid was used as standard [21,34].

### Nitric oxide scavenging activity assay

About 4 ml sample of the extract was taken in a tube and 1 ml of sodium nitroprusside (5 mm in phosphate buffered saline) solution was added into the test tubes. It was then incubated for 2 h at 30°C to complete the reaction. 2 ml of this processed sample was withdrawn from the mixture and mixed with 1.2 ml of Griess reagent (1% sulphanilamide, 0.1% naphthylethylene diamine dihydrochloride in 2% H_3_PO_4_). The absorbance of the chromophore formed during diazotization of nitrite with sulphanilamide, and its subsequent coupling with napthylene diamine was measured at 550 nm [21,34]. Ascorbic acid was used as standard. The percentage (%) inhibition was calculated using the following equation [21,34]:

$$\left\{\left({\text{A}}_{0}-{\text{A}}_{1}\right)/{\text{A}}_{0}\right\}\times 100$$

Where, A_0_ is the absorbance of the control standard, while A_1_ is the absorbance of the extract.

### Statistical analysis of data

All the data collected were analyzed using the Statistical Package for Social Sciences (SPSS) version 17 (SPSS Inc., IL, USA). Results were presented in tables and figures. Continuous variables were presented as mean ± standard deviation. The analysis of variance test was used to compare the mean values for three or more continuous variables representing various different groups in order to establish any level of statistically significant relationship. For all tests, a p-value < 0.05 was considered statistically significant.

## Results

The physicochemical property of the crude aqueous leafy extract of *M. indica* reveals that it has a non pungent, non choking smell and is dark in color. It is also sticky to touch, soluble in water and slightly acidic with a pH of 6.9.

The qualitative phytochemical analysis screening of the crude aqueous leafy extract of *M. indica* revealed the presence of phytochemicals such as tannins, alkaloids, saponins, flavonoids, cardiac glycosides and phenols. The quantitative phytochemical screening also showed the amount of the some of the phytochemicals present in the extract (Table 1).

**Table 1.** Qualitative and quantitative phytochemical screening of the crude aqueous leafy extract of *Mangifera indica*.

Phytochemical constituents	Inferences	Concentration amount (mean ± SD) in mg per 100 g of the *M. indica* crude aqueous leafy extract
Alkaloids	Detectable	12.35 ± 0.26 mg per 100 g
Saponins	Detectable	19.45 ± 0.40 mg per 100 g
Tannins	Detectable	24.42 ± 0.17 mg per 100 g
Cardiac glycosides	Detectable	32.52 ± 0.61 mg per 100 g
Flavonoids	Detectable	37.15 ± 0.37 mg per 100 g
Phenols	Detectable	21.90 ± 0.12 mg per 100 g
Terpenoids	Undetectable	–
Phlobatanins	Undetectable	–
Anthraquinones	Undetectable	–
Steroids	Undetectable	–

SD: Standard deviation.

### Acute toxicity assessment

The acute oral toxicity testing to determine the acute median lethal dose (LD_50_) for the crude aqueous leafy extract of *M. indica* in the mouse model was done using the method described by the OECD in the year 2000 at Paris, France. There was no any observed mortality recorded following the acute oral administration of *M. indica* up to the dose of 5000 mg/kg orally. The crude aqueous leafy extract of *M. indica* has a very wide TI value that is more than 20; with an acute median lethal dose (LD_50_) of more than 5000 mg/kg and a median effective dose (ED_50_) of 250 mg/kg (which was the minimum effective dose that produced an observed significant hepatoprotective, ameliorative and antioxidant effects in at least 50% of the mice used for the acute acetaminophen (paracetamol)-induced hepatotoxicity testing).

### Hepatoprotectiveness evaluation

**Figure 1.**
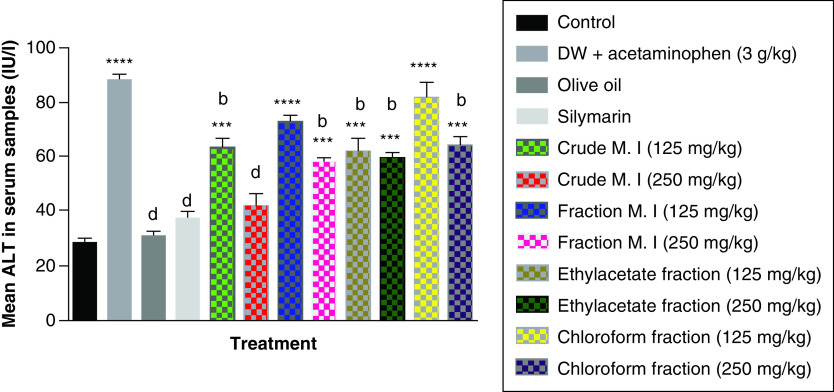
Effect of *Mangifera indica* on alanine transaminase in acute acetaminophen-induced hepatotoxicity. Data represented as mean ± SEM (n = 5). ***p < 0.001 statistically significant compared with control; ****p < 0.0001 statistically significant compared with control; ^b^p < 0.01 statistically significant compared with acetaminophen group; ^d^p < 0.0001 statistically significant compared with acetaminophen-treated group (one-way ANOVA followed by Tukey’s multiple comparison test). ANOVA: Analysis of variance; M. I: *M. indica*; SEM: Standard error of the mean.

Furthermore, acute acetaminophen overdose caused statistically significant increase (p < 0.0001) in the liver enzyme ALT compared with the control group. The effects of acute acetaminophen overdose could also be seen in the presence of different fractions of the leafy extract of *M. indica*-treated mouse groups with acute acetaminophen-induced hepatotoxicity as they were statistically significant compared with the control group (p < 0.001, 0.0001). The different fractions of *M. indica* at the dose of 250 mg/kg was not significantly superior (p > 0.05) compared with the control group but was able to cause significant reduction (p < 0.0001) of the serum ALT liver enzymes when compared with the acetaminophen-treated group. Compare to the doses of 125 mg/kg of the crude aqueous leafy extract of *M. indica*, the different fractions of the leafy extract at the dose of 250 mg/kg of the fractionated aqueous leafy extract, 125 and 250 mg/kg of the ethyl acetate fraction and 250 mg/kg of the petroleum ether-chloroform fraction also produced a significant reduction (p < 0.01) of the serum ALT enzymes compared with the acetaminophen-treated group. The silymarin group was not significantly superior (p > 0.05) compared with the control group but was able to produce a statistically significant reduction (p < 0.0001) in ALT compared with the acetaminophen-treated group (Figure 1).

**Figure 2.**
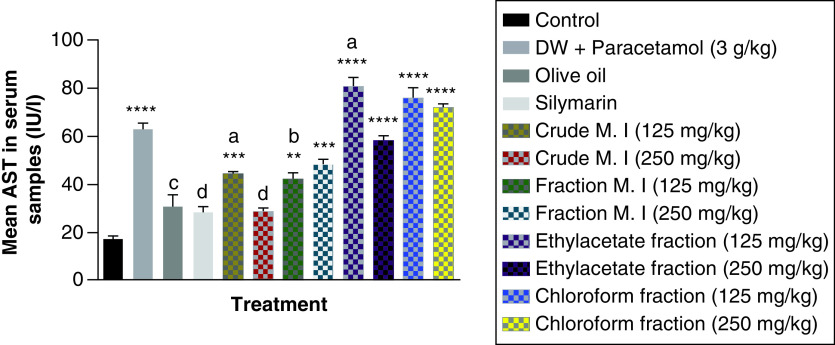
Effect of *M. indica* on aspartate transaminase in acute acetaminophen-induced hepatotoxicity. Data represented as mean ± SEM (n = 5). **p < 0.01 statistically significant compared with control; ***p < 0.001 statistically significant compared with control; ****p < 0.0001 statistically significant compared with control; ^a^p < 0.05 statistically significant compared with acetaminophen group; ^b^p < 0.01 statistically significant compared with acetaminophen group; ^c^p < 0.001 statistically significant compared with acetaminophen group; ^d^p < 0.0001 statistically significant compared with acetaminophen-treated group (one-way ANOVA followed by Tukey’s multiple comparison test). ANOVA: Analysis of variance; M. I: *M. indica*; SEM: Standard error of the mean.

In addition, acute acetaminophen overdose caused statistically significant increase (p < 0.0001) in the liver enzyme AST compared with the control group. The effects of acute acetaminophen overdose could also be seen in the presence of different fractions of the leafy extract of *M. indica*-treated mouse groups with acute acetaminophen-induced hepatotoxicity as they were statistically significant compared with the control group (p < 0.01, 0.001, 0.0001). The different fractions of *M. indica* at the dose of 250 mg/kg was not significantly superior (p > 0.05) compared with the control group but was able to cause significant reduction (p < 0.0001) of the serum AST liver enzymes when compared with the acetaminophen-treated group. Compare to the doses of 125 mg/kg of the crude aqueous leafy extract of *M. indica*, the different fractions of the leafy extract at the dose of 125 mg/kg of the fractionated aqueous leafy extract and 125 mg/kg of the ethyl acetate fraction also produced a significant reduction (p < 0.05, 0.01) of the serum AST enzymes compared with the acetaminophen-treated group. The silymarin group was not significantly superior (p > 0.05) compared with the control group but was able to produce a statistically significant reduction (p < 0.0001) in AST compared with the acetaminophen-treated group (Figure 2).

**Figure 3.**
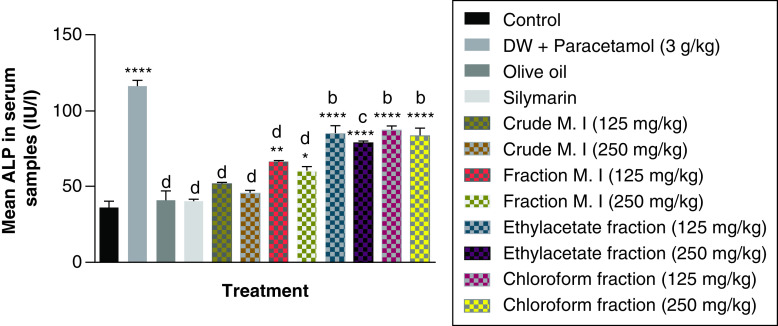
Effect of *M. indica* on alkaline phosphatase in acute acetaminophen-induced hepatotoxicity. Data represented as mean ± SEM (n = 5). *p < 0.05 statistically significant compared with control; **p < 0.01 statistically significant compared with control; ****p < 0.0001 statistically significant compared with control; ^b^p < 0.01 statistically significant compared with acetaminophen group; ^c^p < 0.001 statistically significant compared with acetaminophen group; ^d^p < 0.0001 statistically significant compared with acetaminophen-treated group (one-way ANOVA followed by Tukey’s multiple comparison test). ANOVA: Analysis of variance; M. I: *M. indica*; SEM: Standard error of the mean.

It was observed that acute acetaminophen overdose caused statistically significant increase (p < 0.0001) in the liver enzyme ALP compared with the control group. The effects of acute acetaminophen overdose could also be seen in the presence of different fractions of the leafy extract of *M. indica*-treated mouse groups with acute acetaminophen-induced hepatotoxicity as they were statistically significant compared with the control group (p < 0.05, 0.001, 0.0001). The different fractions of *M. indica* at the doses of 125 and 250 mg/kg were not significantly superior (p > 0.05) compared with the control group but was able to cause significant reduction (p < 0.0001) of the serum ALP enzymes when compared with the acetaminophen-treated group. The different fractions of the leafy extract of *M. indica* at the doses of 125 and 250 mg/kg of the fractionated aqueous leafy extract, 125 and 250 mg/kg of the ethyl acetate fraction and 125 and 250 mg/kg of the petroleum ether-chloroform fraction also produced significant reduction (p < 0.01, 0.001, 0.0001) of the serum ALP enzymes compared with the acetaminophen-treated group. The silymarin group was not significantly superior (p > 0.05) compared with the control group but was able to produce a statistically significant reduction (p < 0.0001) in ALP compared with the acetaminophen-treated group (Figure 3).

**Figure 4.**
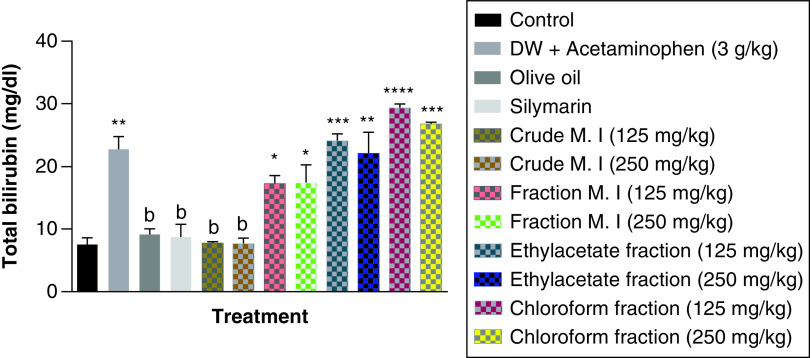
Effect of *M. indica* on total bilirubin levels in acute acetaminophen-induced hepatotoxicity. Data represented as mean ± SEM (n = 5). *p < 0.05 statistically significant compared with control; **p < 0.01 statistically significant compared with control; ***p < 0.001 statistically significant compared with control; ****p < 0.0001 statistically significant compared with control; ^b^p < 0.01 statistically significant compared with acetaminophen-treated group (one-Way ANOVA followed by Tukey’s multiple comparison test). ANOVA: Analysis of variance; M. I: *M. indica*; SEM: Standard error of the mean.

Acute acetaminophen overdose caused statistically significant increase (p < 0.01) in the total bilirubin levels compared with the control group. The effects of acute acetaminophen overdose could also be seen in the presence of different fractions of the leafy extract of *M. indica*-treated mouse groups with acute acetaminophen-induced hepatotoxicity as they were statistically significant compared with the control group (p < 0.05, 0.01, 0.001, 0.0001). The crude aqueous leafy extract of *M. indica* at the doses of 125 and 250 mg/kg was not statistically significant (p > 0.05) compared with the control group, but was able to cause a statistically significant reduction (p < 0.01) in the serum total bilirubin levels when compared with the acetaminophen-treated group. The different fractions of the leafy extract of *M. indica* at the doses of 125 and 250 mg/kg of the fractionated aqueous leafy extract and 125 and 250 mg/kg of the ethyl acetate fraction and 125 and 250 mg/kg of the petroleum ether-chloroform fraction do not produce statistically significant difference (p > 0.05) on the serum total bilirubin levels compared with the acetaminophen-treated group. The silymarin group was not significantly superior (p > 0.05) compared with the control group but was able to produce a statistically significant reduction (p < 0.01) compared with the acetaminophen-treated group (Figure 4).

**Figure 5.**
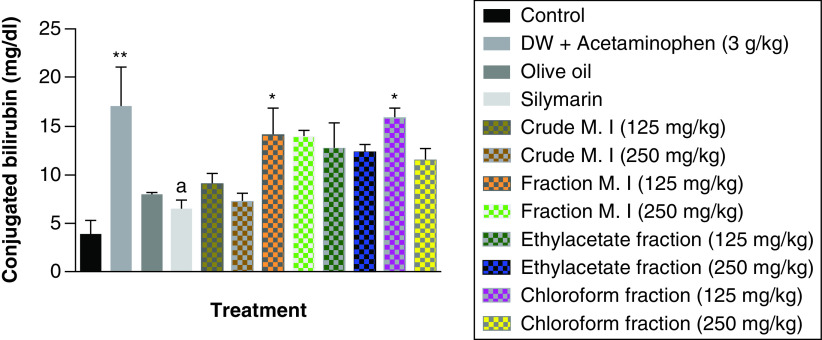
Effect of *M. indica* on conjugated bilirubin levels in acute acetaminophen-induced hepatotoxicity. Data represented as mean ± SEM (n = 5). *p < 0.05 statistically significant compared with control; **p < 0.01 statistically significant compared with control; ^a^p < 0.05 statistically significant compared with acetaminophen-treated group (one-way ANOVA followed by Tukey’s multiple comparison test). ANOVA: Analysis of variance; M. I: *M. indica*; SEM: Standard error of the mean.

It was observed that acute acetaminophen overdose caused statistically significant increase (p < 0.01) in the conjugated bilirubin levels compared with the control group. The effects of acute acetaminophen overdose were observed in the presence of different fractions of the leafy extract of *M. indica*-treated mouse groups with acute acetaminophen-induced hepatotoxicity as 125 mg/kg fractionated aqueous leafy extract and fractionated petroleum ether-chloroform extract were statistically significant (p < 0.05) compared with the control. None of the *M. indica* fractions produced any statistically significant reduction (p > 0.05) in the conjugated bilirubin levels compared with the acetaminophen-treated group. The silymarin group, the 125 mg/kg of the crude aqueous leafy extract group or the 250 mg/kg of the crude aqueous leafy extract group was not statistically significant (p > 0.05) compared with the control group but was able to produce statistically significant reduction (p < 0.05) compared with the acetaminophen-treated group (Figure 5).

**Figure 6.**
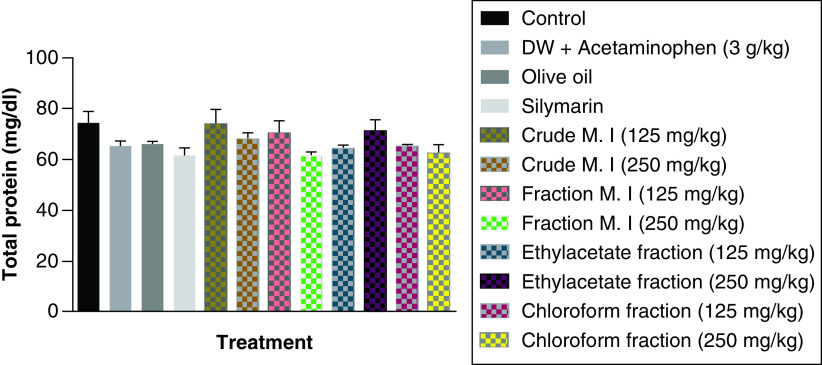
Effect of *M. indica* on serum total protein levels in acute acetaminophen-induced hepatotoxicity. Data represented as mean ± SEM (n = 5). p > 0.05 not statistically significant compared with either control group or acetaminophen-treated group (one-way ANOVA followed by Tukey’s multiple comparison test). ANOVA: Analysis of variance; M. I: *M. indica*; SEM: Standard error of the mean.

However, it was observed that acute acetaminophen overdose did not have any statistically significant effect (p > 0.05) on the serum total protein levels compared with the control group. None of the of the *M. indica* fractions also had any statistically significant effect (p > 0.05) on the serum total protein levels compared with the acetaminophen-treated group. The silymarin group was not statistically significant (p > 0.05) compared with the control group and had no significant effect (p > 0.05) on the serum total protein levels compared with the acetaminophen-treated group (Figure 6).

**Figure 7.**
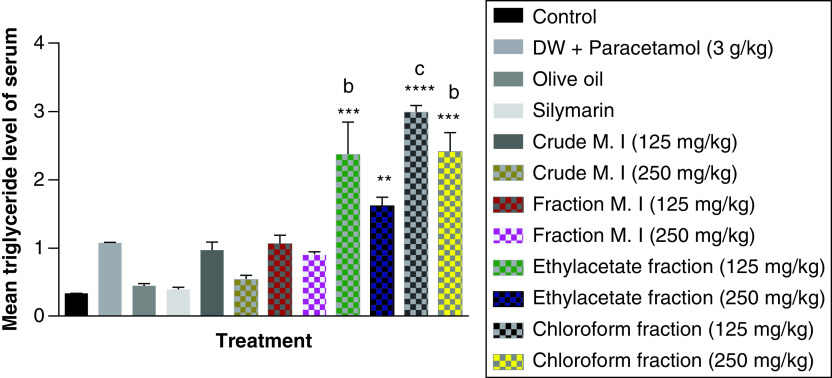
Effect of *M. indica* on serum triglyceride levels in acute acetaminophen-induced hepatotoxicity. Data represented as mean ± SEM (n = 5). **p < 0.01 statistically significant compared with control; ***p < 0.001 statistically significant compared with control; ****p < 0.0001 statistically significant compared with control; ^b^p < 0.01 statistically significant compared with acetaminophen-treated group; ^c^p < 0.001 statistically significant compared with acetaminophen-treated group (one-way ANOVA followed by Tukey’s multiple comparison test). ANOVA: Analysis of variance; M. I: *M. indica*; SEM: Standard error of the mean.

In this study, acute acetaminophen overdose did not have any statistically significant effect on the serum triglyceride levels (p > 0.05) compared with the control group. The fractions of ethyl acetate (125 and 250 mg/kg) and petroleum ether-chloroform (125 and 250 mg/kg) of the *M. indica* produced a statistically significant increase of the serum triglyceride levels compared with the control group (p < 0.01, 0.001). The fractions of ethyl acetate (125 mg/kg) and petroleum ether-chloroform (125 and 250 mg/kg) of the *M. indica* had a statistically significant increase on the serum triglyceride levels compared with the acetaminophen-treated group (p < 0.01, 0.001). The silymarin group was not statistically significant (p > 0.05) compared with the control group and had no statistically significant effect on the serum triglyceride levels (p > 0.05) compared with the acetaminophen-treated group (Figure 7).

**Figure 8.**
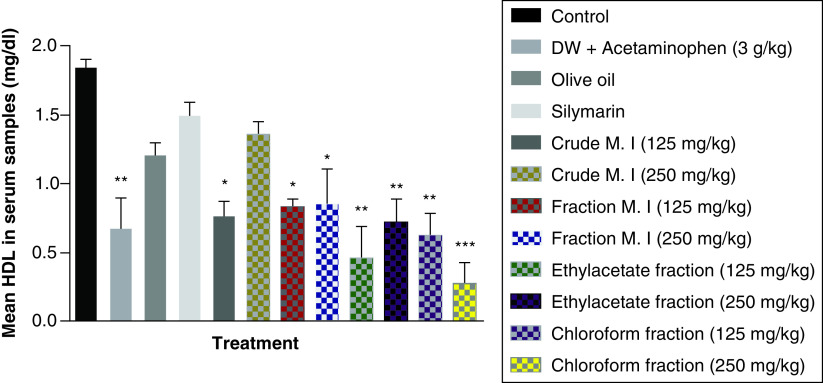
Effect of *M. indica* on high density lipoprotein cholesterol levels in acute acetaminophen-induced hepatotoxicity. Data represented as mean ± SEM (n = 5). *p > 0.05 not statistically significant compared with control; **p < 0.01 statistically significant compared with control; ***p < 0.001 statistically significant compared with control (one-way ANOVA followed by Tukey’s multiple comparison test). ANOVA: Analysis of variance; M. I: *M. indica*; SEM: Standard error of the mean.

Acute acetaminophen overdose produced a statistically significant decrease in the serum high density lipoprotein (HDL) cholesterol levels (p < 0.01) compared with the control group. All the different fractions of the leafy extract of *M. indica* produced a statistically significant decrease of the serum HDL cholesterol levels compared with the control group (p < 0.01, 0.001) except for the fractionated aqueous leafy extract (125 and 250 mg/kg) that was not statistically significant (p > 0.05) compared with the control group. None of the fractions of the leafy extract of *M. indica* had any statistically significant effect on the serum HDL cholesterol levels compared with the acetaminophen-treated group (p > 0.05) and silymarin group had no effect (p > 0.05) compared with the acetaminophen-treated group (Figure 8).

**Figure 9.**
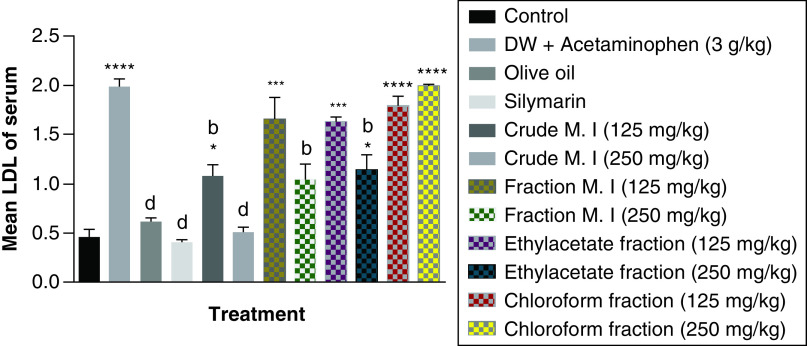
Effect of *M. indica* on low density lipoprotein cholesterol levels in acute acetaminophen-induced hepatotoxicity. Data represented as mean ± SEM (n = 5). *p < 0.05 statistically significant compared with control; ***p < 0.001 statistically significant compared with control; ****p < 0.0001 statistically significant compared with control; ^b^p < 0.01 statistically significant compared with acetaminophen-treated group; ^d^p < 0.0001 statistically significant compared with acetaminophen-treated group (one-way ANOVA followed by Tukey's multiple comparison test). ANOVA: Analysis of variance; M. I: *M. indica*; SEM: Standard error of the mean.

Furthermore, acute acetaminophen overdose produced a statistically significant increase (p < 0.0001) in low density lipoprotein [LDL] cholesterol levels compared with the control group. The effects of acute acetaminophen overdose were seen in the presence of different fractions of the leafy extract of *M. indica* treated mouse groups with acute acetaminophen-induced hepatotoxicity as they were statistically significant compared with the control group (p < 0.05, 0.001, 0.0001). The crude aqueous leafy extract (at the dose of 125 mg/kg) and fractionated aqueous leafy extract (at the dose of 250 mg/kg) of *M. indica* were not statistically significant (p > 0.05) compared with the control group but was able to produce statistically significant reduction (p < 0.01, 0.0001) of the serum LDL cholesterol levels when compared with the acetaminophen-treated group. The ethyl acetate fraction (at the dose of 250 mg/kg) also produced a statistically significant decrease (p < 0.01) in the serum LDL cholesterol levels compared with the acetaminophen-treated group. The silymarin group did not show statistically significant reduction (p > 0.05) in the serum LDL cholesterol levels compared with the control group but was able to exhibit statistically significant reduction (p < 0.0001) compared with the acetaminophen-treated group (Figure 9).

**Figure 10.**
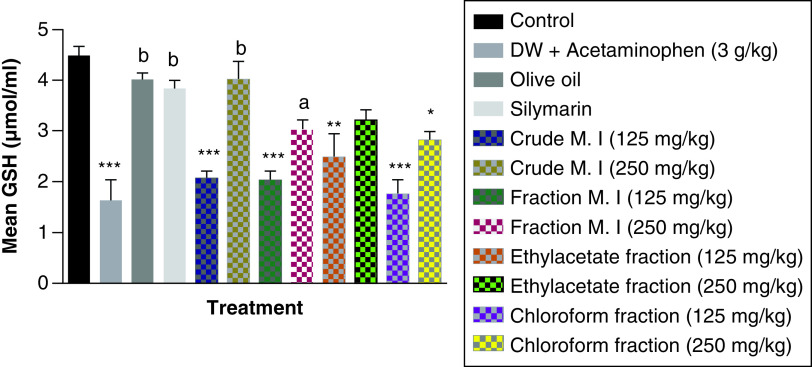
Effect of *Mangifera indica* on the levels of glutathione-reduced form [GSH] in acute acetaminophen-induced hepatotoxicity. Data represented as mean ± SEM (n = 5). *p < 0.05 statistically significant compared with control; **p < 0.01 statistically significant compared with control; ***p < 0.001 statistically significant compared with control; ^a^p < 0.05 statistically significant compared with acetaminophen-treated group; ^b^p < 0.01 statistically significant compared with acetaminophen-treated group (one-way ANOVA followed by Tukey’s multiple comparison test). ANOVA: Analysis of variance; M. I: *M. indica*; SEM: Standard error of the mean.

In this study, acute acetaminophen overdose produced a statistically significant decrease in the levels of glutathione-reduced form (GSH) among the acetaminophen-treated group (p < 0.001) compared with the control group. These statistically significant reductions could be seen in all the different *M. indica* groups at the dose of 125 mg/kg each of the crude aqueous leafy extract, fractionated aqueous leafy extract, ethyl acetate fraction and petroleum ether-chloroform fraction. Also, the crude aqueous leafy extract, fractionated aqueous leafy extract, ethyl acetate fraction and petroleum ether-chloroform fraction of *M. indica* at the dose of 250 mg/kg each produced a statistically significant increase (p < 0.01, 0.05, 0.05 and 0.05) in the levels of GSH compared with the acetaminophen-treated group. The silymarin group also had a statistically significant increase (p < 0.01) in their GSH levels compared with the acetaminophen-treated group (Figure 10).

**Figure 11.**
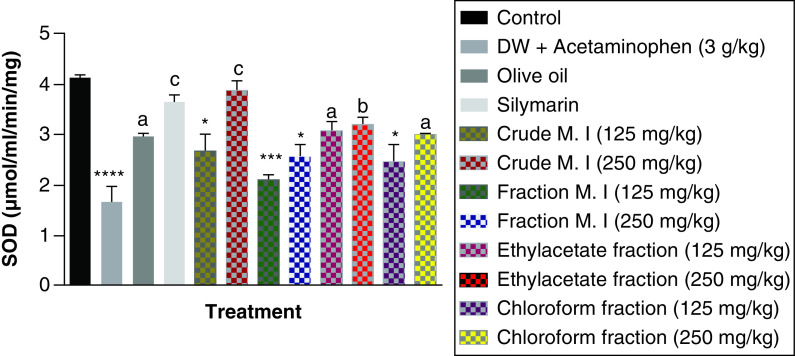
Effect of *M. indica* on superoxide dismutase levels in acute acetaminophen-induced hepatotoxicity. Data represented as mean ± SEM (n = 5). *p < 0.05 statistically significant compared with control; ***p < 0.001 statistically significant compared with control; ****p < 0.0001 statistically significant compared with control; ^a^p < 0.05 statistically significant compared with acetaminophen-treated group; ^b^p < 0.01 statistically significant compared with acetaminophen-treated group; ^c^p < 0.001 statistically significant compared with acetaminophen-treated group (one-way ANOVA followed by Tukey’s multiple comparison test). ANOVA: Analysis of variance; M. I: *M. indica*; SEM: Standard error of the mean.

Also, acute acetaminophen overdose produced a statistically significant reduction in the superoxide dismutase (SOD) levels of the acetaminophen-treated group (p < 0.0001) compared with the control group. The result also showed no statistically significant reduction in the effect of ethyl acetate fraction on SOD levels compared with the control group (p > 0.05). The crude aqueous leafy extract (125 mg/kg), fractionated aqueous leafy extract (at 125 and 250 mg/kg) and petroleum ether-chloroform fraction (125 mg/kg) of *M. indica* exhibited a statistically significant effect on the SOD levels (p < 0.05, 0.001) compared with the control group. The crude aqueous leafy extract (250 mg/kg), fractionated aqueous leafy extract (at 125 and 250 mg/kg) and petroleum ether-chloroform fraction (250 mg/kg) of the leafy extract of *M. indica* produce statistically significant increase in the SOD levels (p < 0.001, 0.001, 0.05) compared with the acetaminophen-treated group. The silymarin group also exhibited a statistically significant increase (p < 0.001) in their SOD levels compared with the acetaminophen-treated group (Figure 11).

**Figure 12.**
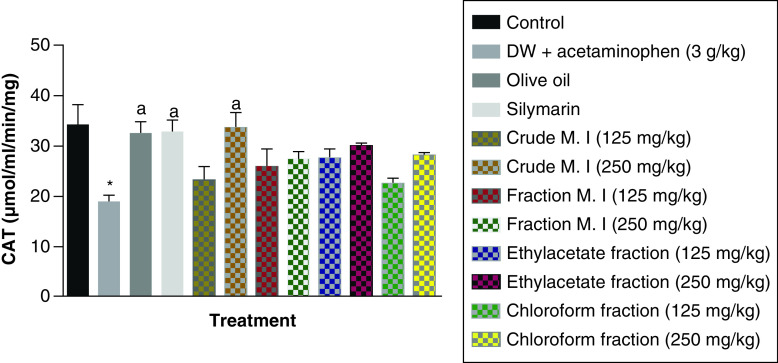
Effect of *M. indica* on catalase levels in acute acetaminophen-induced hepatotoxicity. Data represented as mean ± SEM (n = 5). *p < 0.05 statistically significant compared with control; ^a^p < 0.05 statistically significant compared with acetaminophen-treated group (one-way ANOVA followed by Tukey’s multiple comparison test). ANOVA: Analysis of variance; M. I: *M. indica*; SEM: Standard error of the mean.

In this study, it was observed that acute acetaminophen overdose produced a statistically significant reduction in the levels of catalase (CAT) antioxidant enzyme of the acetaminophen-treated group (p < 0.05) compared with the control group. All of the different separating medium fractions of *M. indica* did not have any statistically significant effect (p > 0.05) on acute acetaminophen-induced hepatotoxicity. However, only the 250 mg/kg dose of the crude aqueous leafy extract produced a statistically significant increase (p < 0.05) in the CAT antioxidant enzyme levels compared with the acetaminophen-treated group. The silymarin group also had a statistically significant increase (p < 0.05) in their CAT antioxidant enzyme levels compared with the acetaminophen-treated group (Figure 12).

**Figure 13.**
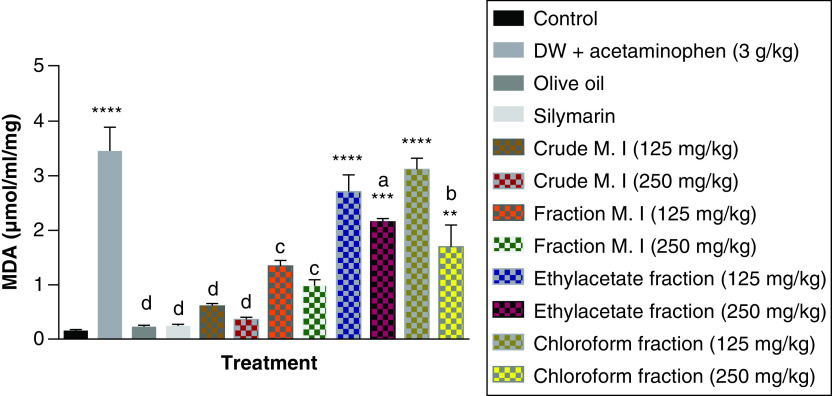
Effect of *M. indica* on malondialdehyde levels in acute acetaminophen-induced hepatotoxicity. Data represented as mean ± SEM (n = 5). **p < 0.01 statistically significant compared with control; ***p < 0.001 statistically significant compared with control; ****p < 0.0001 statistically significant compared with control; ^a^p < 0.05 statistically significant compared with acetaminophen-treated group; ^b^p < 0.01 statistically significant compared with acetaminophen-treated group; ^c^p < 0.001 statistically significant compared with acetaminophen-treated group; ^d^p < 0.0001 statistically significant compared with acetaminophen-treated group (one-way ANOVA followed by Tukey’s multiple comparison test). ANOVA: Analysis of variance; M. I: *M. indica*; SEM: Standard error of the mean.

Acute acetaminophen overdose produced a statistically significant increase (p < 0.0001) in the MDA levels compared with the control group. The effects of the ethyl acetate fraction and petroleum ether-chloroform fraction were statistically significant (p < 0.01, 0.001, 0.0001) on the MDA levels compared with the control group. But, the crude aqueous leafy extract and fractionated aqueous leafy extract were not statistically significant (p > 0.05) compared with the control group. Both the crude aqueous leafy extract and fractionated aqueous leafy extract of *M. indica* (at the doses of 125 and 250 mg/kg each) produced statistically significant reduction in the MDA levels (p < 0.001, 0.0001) compared with the acetaminophen-treated group. The 250 mg/kg dose of the ethyl acetate fraction and 250 mg/kg dose of the petroleum ether-chloroform fraction also had statistically significant reduction in the MDA levels (p < 0.05, 0.01) compared with the acetaminophen-treated group. The silymarin group was not statistically significant (p > 0.05) compared with the control group but was able to cause statistically significant reduction (p < 0.0001) in the MDA levels compared with the acetaminophen-treated group (Figure 13).

## Discussion

Drug-induced hepatotoxicity is still a major and significant public health problem to the healthcare professionals (physicians and nurses) as well as the healthcare products regulatory bodies, including the WHO and US FDA. The challenges posed by this pathologic condition affect mainly the development of new pharmaceutical drugs and the withdrawal of promising pharmaceutical drugs from the pharmaceutical market.

The generation of reactive oxygen species (ROS), proinflammatory mediators and direct action on the cellular organelles of hepatocytes had been reported as some of the mechanisms underlying the induction, onset and occurrence of drug-induced hepatotoxicity [12,16].

The hepatotoxicants that act by formation of ROS include acetaminophen [12], carbon tetrachloride [13], amodiaquine [14], halothane [15], isoniazid [12,16] allyl alcohol and bromobenzene. They undergo biotransformation to chemically reactive toxic metabolites which can covalently bind to crucial cellular macromolecules thus inactivating, inhibiting or blocking critical cellular functions [9].

Davidson and Eastham were the first to report that acetaminophen was hepatotoxic in overdose [33]. They described two individuals who developed hepatotoxicity following acute acetaminophen overdose and died on the third day following fulminant hepatic failure. Acetaminophen has been confirmed as a lethal hepatotoxic substance at very high single oral dose of 3 g/kg body weight [12] which was the rationale behind its use in this study and also because it is a moderately potent analgesic and antipyretic agent that human beings ingest to relieve mild-to-moderate pains and low-to-intermediate grade fevers [12,16]. Early researches to understanding oxidative stress in acetaminophen-induced hepatotoxicity focused on iron-mediated oxidative stress (Fenton mechanism). This mechanism is initiated by cellular superoxide formation and its dismutation to form increased hydrogen peroxide. Superoxide may be formed by multiple mechanisms including uncoupling of cytochrome P-4502E1 or other enzymes and mitochondria, or activation of NADPH oxidase [12,16]. Since glutathione is depleted by the metabolite N-acetyl-p-benzoquinone imine (NAPQI) in acetaminophen-induced hepatotoxicity and glutathione is the cofactor for glutathione peroxidase detoxification of peroxides, a major mechanism of peroxide detoxification is compromised in acetaminophen-induced toxicity. Thus, glutathione depletion may be expected to lead to increased intracellular peroxide levels and increased oxidative stress via a Fenton mechanism. This mechanism involves the reduction of peroxide by ferrous ions forming the highly reactive hydroxyl radical which may in turn oxidize lipids leading to initiation of lipid peroxidation as well as oxidation of proteins and nucleic acids [12,16]. Microscopic examination of liver sections from acetaminophen-induced hepatitis affected individuals indicated fulminating hepatic necrosis. The necrosis was primarily in the centrilobular areas. Eosinophilic degeneration of the cells together with pyknosis of nuclear material was observed in these hepatocytes. Vacuolization and early degenerative changes were observed in the more peripheral cells surrounding the portal areas. A mild polymorphonuclear leukocytic infiltration occurred in both cases. These changes indicated fulminating necrosis confined primarily to the hepatocytes in the centrilobular regions of the liver [21]. Acetaminophen overdose is characterized by principal clinical symptoms such as development of nausea and vomiting within 2–3 h of overdose ingestion, followed by severe abdominal pain in the right upper quadrant. Liver dysfunction occurred within 24 h and reached a maximum state in approximately 3–4 days after ingestion [21]. The three different mechanisms that have been suggested to account for the increased level of ROS formation in acute acetaminophen-induced hepatotoxicity are: uncoupling of CYP2E1 or other enzymes, activation of NADPH oxidase, mitochondrial uncoupling and glutathione depletion [12,16].

Liver injury leads to disturbance in the transport functions of hepatocytes resulting in leakage intracellular enzymes through plasma membrane breakdown which results to increase level of ALT, AST, ALP, gamma glutamyl transferase and bilirubin in the systemic circulation plasma preclinically and clinically [12,16,18–20].

This study was designed to investigate the hepatoprotective, ameliorative and antioxidant effects of *M. indica* crude aqueous leafy extract and its different separating medium fractions against acute acetaminophen-induced hepatotoxicity in a mice model. The acute toxicity profile for the crude aqueous leafy extract of *M. indica* in the mice model for oral dose of up to 5000 mg/kg revealed no associated mortality/lethality; indicating that this extract is quite safe orally with a very wide TI value more than 20; an acute median lethal dose (LD_50_) of more than 5000 mg/kg, and a median effective dose (ED_50_) of 250 mg/kg. In this study, the hepatoprotective and ameliorative effects of *M. indica* was evaluated through its observed influence and activity on the liver enzymes. The assessment of hepatocellular injury is mainly done by determining the levels of hepatic enzyme markers such as AST, ALT, ALP and bilirubin in the peripheral blood. Necrosis or membrane damage releases these hepatic enzymes into the systemic (peripheral) circulation and hence it can be measured in the serum. High level of AST indicates liver damage that involves the mitochondrial organelles, such as that caused by viral hepatitis as well as other pathologic conditions such as myocardial infarction and skeletal muscle injury. The AST enzyme catalyzes the conversion of oxaloacetate to aspartate via transaminase reaction. Furthermore, the ALT enzyme is more specific to the liver, and is thus a better marker for detecting early liver injury. The ALT enzyme is exclusively a cytoplasmic enzyme which can easily leak-out into the systemic (peripheral) circulation following damage to hepatocytes' plasma membranes. The ALT enzyme catalyzes the conversion of pyruvate to alanine via transaminase reaction. Elevated serum levels of these hepatic enzymes (AST and ALT) are indicative of hepatocellular damage/leakage and loss of functional integrity of hepatocyte’s cell membrane in the liver [12,16,18–20].

Serum ALP and conjugated bilirubin levels, on the other hand, are related to the functional integrity of the hepatobiliary tracts. Elevation of the serum ALP level is due to increase synthesis or cholestatic obstruction to the free flow of biliary tract contents in the presence of increased biliary pressure [12,16,18–20].

However, this current study was designed to evaluate the hepatoprotective, ameliorative and antioxidant potentials of *M. indica* crude aqueous leafy extract. The results showed that *M. indica* was able to significantly reduce the levels of liver enzymes that were increased by acute acetaminophen-induced hepatotoxicity compared with the acetaminophen-treated group. The effects of *M. indica* were mainly observed in the crude aqueous leafy extract, fractionated aqueous leafy extract and ethyl acetate fractions. The petroleum ether-chloroform fraction also exhibited varying effects on the liver enzymes but cannot be compared with the prominent (significant) effects of the crude aqueous leafy extract.

On the other biochemical parameters such as total bilirubin and conjugated bilirubin levels; this study also revealed the ameliorative effects of crude aqueous leafy extract of *M. indica* and its different separating medium fractions by significantly reducing acute acetaminophen-induced hepatotoxicity. According to this study, the effect of *M. indica* was not seen on serum totals protein level just in the same way the effect of the hepatotoxicant (acetaminophen) was not seen on serum total protein levels. The effects of *M. indica* on lipid profile revealed that the crude aqueous leafy extract and its different separating medium fractions reduced LDL levels, increased HDL levels, but did not have any statistically significant effect on triglyceride levels compared with the acetaminophen-treated group.

The effect of *M. indica* on antioxidant parameters also showed that the extract increased the GSH, SOD and CAT levels compared with the acetaminophen-treated group. But *M. indica* reduced the MDA levels compared with the acetaminophen-treated group thereby ameliorating and reversing the effect of the hepatotoxicant (acetaminophen) on liver tissue. In this study, our finding also showed that the olive oil used as solvent of the petroleum ether-chloroform fraction had no conflicting effect when the olive oil group was compared with the control group in the biochemical and antioxidant parameters. According to this study, the standard hepatoprotective herbal drug-silymarin, was able to ameliorate and prophylactically prevent the effect of the hepatotoxicant (acetaminophen) in almost all the biochemical and antioxidant parameters except for the serum total protein and triglyceride levels where the hepatotoxicant (acetaminophen) did not have any statistically significant influence.

Earlier reports had shown that the bark of *M. indica* tree possesses significant antioxidant properties [28,29]. Also, this study further corroborated the antioxidant effect of the crude aqueous leafy extract of *M. indica* plant, as it significantly attenuated and reversed the pro-oxidant activity of lipofundin-induced oxidative stress. There are other medicinal values of the *M. indica* plant, as Prasad and Kalra demonstrated the hepatoprotective effect of the seed kernel extract of *M. indica* fruits [30].

One of the known mechanisms of hepatotoxicity action of acetaminophen overdose has been the generation of ROS [12,16] among many other mechanisms of hepatotoxicity. The counteracting effect of *M. indica* due to its antioxidant properties may be the underlying mechanism of hepatoprotective action for its crude aqueous leafy extract against acute acetaminophen (paracetamol)-induced hepatotoxicity as demonstrated and observed in this study. The *M. indica* extract used in this study contains tannins, alkaloids, saponins, flavonoids, cardiac glycosides and phenols which were confirmed by phytochemical analysis of the crude aqueous leafy extract. The flavonoids, saponins, alkaloids and tannins phytochemical constituent groups are well recognized for their hepatoprotective and antioxidant actions [30–32,35,36]. As previous studies also demonstrated that saponins, alkaloids, flavonoids and tannins were the active phytochemical constituents of *M. indica* plant that possesses antioxidant properties, free radical scavenging ability and inhibition of lipid peroxidation reactions [30–32,35,36].

## Conclusion

In conclusion, the experimental findings from this study suggest that the crude aqueous leafy extract of *M. indica* plant possesses hepatoprotective, ameliorative and antioxidant effects. These activities are possibly mediated through the induction of antioxidant enzymes to prevent the occurrence of oxidative stress damage or most likely through the inhibition of proinflammatory mediators which are being produced by acute acetaminophen (paracetamol)-induced hepatotoxicity. The observed results justify the traditional use of this medicinal plant extract as a prophylacting hepatoprotective agent for the prevention of toxin-induced hepatitis. Finally, it can also be deduced that the various active phytochemical constituents of this crude aqueous leafy extract altogether, contribute better to its observed overall hepatoprotective, ameliorative and antioxidant effects than these various groups of its different separating medium fractional components that have sub-optimal biologic activity against acetaminophen (paracetamol)-induced hepatotoxicity.

## Recommendation, applications & future research perspective

The observed results in this study justify the traditional use of this medicinal crude aqueous leafy extract from *M. indica* plant as a prophylacting hepatoprotective agent for the prevention of toxin-induced hepatitis. We recommend that a subacute, subchronic and chronic toxicity studies in a mouse model be carried out on the crude aqueous leafy extract of *M. indica* plant in order to ascertain and establish its long term safety profile. If the preclinical long-term safety profile for the crude aqueous leafy extract of *M. indica* plant in a mouse model is found to be quite favorable; the clinical trial of this herbal extract can be done by applying for the investigation of new drug status with the national and international drug regulatory agencies. The complete pharmaceutical isolation, analysis and characterization profile of the main pharmacologically active hepatoprotective and antioxidant phytochemical constituent(s) of the crude aqueous leafy extract of *M. indica* plant will soon be done experimentally.

**Summary points**

- The experimental findings from this study suggest that the crude aqueous leafy extract of *Mangifera indica* possesses hepatoprotective, ameliorative and antioxidant effects. These activities are possibly mediated through the induction of antioxidant enzymes to prevent the occurrence of oxidative stress damage or most likely through the inhibition of pro-inflammatory mediators which are being produced by acute acetaminophen-induced hepatotoxicity. The observed results justify the traditional use of this medicinal plant extract as a prophylacting hepatoprotective agent for the prevention of toxin-induced hepatitis.
- In addition, it can also be deduced that the various active phytochemical constituents of this crude aqueous leafy extract altogether, contribute better to its observed overall hepatoprotective, ameliorative and antioxidant effects than these various groups of its different separating medium fractional components that have sub-optimal biologic activity against acetaminophen (paracetamol)-induced hepatotoxicity.
- Also, one of the known mechanisms of hepatotoxicity action of acetaminophen overdose has been the generation of reactive oxygen species among many other mechanisms of hepatotoxicity. The counteracting effect of *M. indica* due to its antioxidant properties may be the underlying mechanism of hepatoprotective action for its crude aqueous leafy extract against acute acetaminophen (paracetamol)-induced hepatotoxicity as demonstrated and observed in this study. The *M. indica* extract used in this study contains tannins, alkaloids, saponins, flavonoids, cardiac glycosides and phenols which were confirmed by phytochemical analysis of the crude aqueous leafy extract. The flavonoids, saponins, alkaloids and tannins phytochemical constituent groups are well recognized for their hepatoprotective and antioxidant actions. As previous studies also demonstrated that saponins, alkaloids, flavonoids and tannins were the active phytochemical constituents of *M. indica* plant that possesses antioxidant properties, free radical scavenging ability and inhibition of lipid peroxidation reactions.
